# Practical Notes on Diagnosis and Treatment

**Published:** 1906-09-15

**Authors:** 


### Practical Notes on Diagnosis and Treatment.

**Aspirin in Chorea.**

In severe cases of chorea aspirin is said to give better results than any other remedy. It is given in doses of 40 to 80 grains daily (to children of ten to fifteen years), the amount being reduced as the symptoms become less urgent. Some good effects are noticed as early as the second day. The remedy may be continued for two to three weeks.

**Treatment of Infantile Syphilis.**

Grey powder, three-quarters of a grain thrice daily, gives the best results. Inunction may be used when it is necessary to get the child quickly under the influence of mercury, and liquor hydrargyri perchlor. in doses of 4 to 10 minims where powders cannot be given. Treatment should be continued for two years after all symptoms have disappeared.—*Dr. G. F. Still.*

**Treatment of Lichen Planus.**

To allay the itching, three to five grains of thymol to the ounce of olive oil is an excellent application. For internal treatment the best remedy in most cases is salicin. Another plan for these cases is to order an effervescing mixture with five grains of quinine in each dose. In the more sub-acute cases also salicin is useful; arsenic has much reputation, but is sometimes disappointing; biniodide of mercury sometimes succeeds, and in cases where the general health is broken down, mineral acids with nux vomica should be tried.—*Dr. Radcliffe Crocker.*

**Drugs in Diabetes.**

So long as the diabetic is allowed large quantities of carbohydrate food there is no object in giving opium, for under such circumstances he is hardly, if at all, affected by opium. The action of opium is, however, very powerful in cases in which the glycosuria has already been reduced to a minimum by abstention from carbohydrate food. Salicylic acid, on the other hand, acts best in slight cases of glycosuria and during the time when carbohydrates are permitted. It raises the power of assimilating carbohydrates not inconsiderably. In no case should salicylic acid or the products related to it be used continuously. There is, however, nothing to be said against their employment for a few weeks in the year in slight cases of diabetes.—*Professor von Noorden.*

**Prognosis in Tabes Dorsalis.**

Having progressed up to a certain point, tabes may cease to advance. All the various symptoms and complications, even such things as disease of bone, alterations in joints, etc., may all, or many of them, be transitory. It must not, therefore, be concluded that everybody who begins with tabes is necessarily going to continue to get worse. The patient's symptoms may either progress or the disease may be arrested, and the arrest may occur either at an early period or at a comparatively late stage of the disorder. Nothing much can be done to arrest the progress of tabes. The main thing to do is to improve the general nutrition of the patient. Antisyphilitic remedies are of no use.—*Mr. A. A. Bowlby.*

**Hæmoptysis in Pneumonia.**

Occasionally a pneumonia of the apex may be accompanied by a sharp profuse hæmoptysis.—*Dr. Donald Hood.*

**Mercury in General Paralysis of the Insane.**

General paralysis should be treated by small doses of mercury. If this is commenced in the very early stage a cure may be obtained; the treatment must not be limited, but continued permanently.—*Mr. Jonathan Hutchinson.*

**Protargol in Conjunctivitis.**

In chronic inflammation of the conjunctiva and the edges of the eyelids, protargol, in from 10 to 25 per cent. solution or ointment brushed vigorously over the affected parts produces far quicker and better results than can be obtained by any other method.—*Dr. Maitland Ramsay.*

**Instrumentation in Renal Inadequacy.**

"When a patient comes to you with a history of difficulty in micturition, with a urine of very low specific gravity, with thirst, lumbar aching, dry tongue, and harsh skin, be very wary how you deal with him instrumentally, for these signs mean renal inadequacy, due to back pressure."—*Mr. Jno. Pardoe.*

**To Find the Head of a Tapeworm.**

The worm usually breaks at about 1½ inches from the head, which is thus left as a very slender filament. To facilitate the search, the pan in which the motion is passed should be covered with black crêpe, against which the fragment terminating in the head may be readily seen.—*Dr. J. Kingston Fowler.*

**Purulent Ophthalmia.**

In the treatment of purulent ophthalmia, whether in the adult or in the new-born child, the best results are obtained by brushing the whole conjunctiva once thoroughly with a 2-per-cent. solution of silver nitrate at the onset of the disease, and afterwards applying a 20-per-cent. solution of argyrol every few hours, the frequency of these applications being regulated solely by the amount of the discharge.—*Dr. Maitland Ramsay.*

**Treatment in Cases of Tapeworm.**

The patient should be kept in bed for three or four days and on restricted diet—namely, beef-tea, two pints; Mason's essence, one tin; two rusks; and port wine, four ounces. For the same period two grains of extract of cascara are given thrice daily. On the fourth day (say at 5 a.m.) one ounce of mist. sennæ co. is given, followed at 9 a.m. by a capsule containing five minims of liquid extract of male fern, this dose being repeated at 9.15, 9.30, and 9.45. At eleven o'clock one ounce of mist. sennæ co. is taken. If by one o'clock the worm has not been passed and the head found, the male-fern treatment with the subsequent purgative draught is recommenced. Even a third course may be necessary, but it is not advisable to go beyond this without an interval of a day's rest.—*Dr. J. Kingston Fowler.*

